# Hospital Emergency Management of Emerging Infectious Disease using Instant Communication Technology

**DOI:** 10.1017/S1049023X20000618

**Published:** 2020-05-12

**Authors:** Chih-Hao Lin, Chih-Chia Hsieh, Chih-Hsien Chi

**Affiliations:** Department of Emergency Medicine, National Cheng Kung University Hospital, College of Medicine, National Cheng Kung University, Tainan, Taiwan

**Keywords:** communication, COVID-19, hospital emergency management, infectious disease

To the Editor:

On January 21, 2020, Taiwan reported its first confirmed case of COVID-19 infection.^1^ The case caused a cumulative effect in the LINE (LINE Corp.; Tokyo, Japan) chat group amongst the emergency medicine employees at a tertiary hospital in Taiwan. The participation of emergency physicians in the chat group increased from 81% (39/48) to 100%. The daily number of chat group messages had a ten-fold increase.

Instant communication devices, instead of phones and handheld transceivers, are commonly used as the main communication tool in hospital emergency management nowadays. Innovative applications on electronic equipment, such as LINE or WhatsApp (WhatsApp Inc.; Menlo Park, California USA), could extensively reshape the concept of information management in emergencies.^2^

Several considerations related to using innovative applications to manage the threat of emerging infectious disease are noteworthy. First, the chat group members should be solicited in a planned way. Considering information security and communication efficiency, the composition of chat group membership should preferably be based on functional groups or incident management teams. The operation, planning, logistics, and finance and administration sections ought to have their own independent chat groups. The command staff need a specific chat group that includes the incident commander or unified command, the safety officer, the liaison officer, the public information officer, and the section leaders of the general staff.^3,4^

Second, the messages in the chat groups need to be regulated. While the instant distribution of messages can effectively and extensively deliver information, the group members can be easily overloaded with unorganized, unscheduled pop-out data. In addition, protocols that are under discussion could be mistaken as a confirmed policy. The discussion messages within such groups should use simple words and common terminology to avoid confusion. Newly developed policies and action plans should be updated on a scheduled basis using a specific format of notification.

Third, instant communication devices should be sterilized regularly. Instant communication applications usually operate on electronic devices such as smartphones, tablet computers, and personal computers. The convenience of instant communication and video conferencing may minimize the necessity of face-to-face meetings and therefore lessen the risk of the aerosol or contact transmission of infectious diseases. However, the frequent use of mobile communication devices carries a risk of repetitive cyclic contamination between the hands and face and thus could lead to disease transmission.^5^ Therefore, the chat group members should be regularly notified to sterilize their hands and mobile devices.

In summary, hospital emergency management should be updated with both innovation and technology.

## Author Contributions

Chih-Hao Lin and Chih-Hsien Chi conceived the study. Chih-Hao Lin contributed to acquisition of data and performed statistical analysis. Chih-Hao Lin drafted the manuscript. Chih-Hao Lin, Chih-Chia Hsieh, and Chih-Hsien Chi contributed substantially to its revision. Chih-Hao Lin is the corresponding author who takes responsibility for the paper as a whole.
